# Effect of single dose N-acetylcysteine administration on resting state functional connectivity in schizophrenia

**DOI:** 10.1007/s00213-019-05382-1

**Published:** 2019-11-30

**Authors:** Grant McQueen, Aderlee Lay, John Lally, Anthony S. Gabay, Tracy Collier, David J. Lythgoe, Gareth J. Barker, James M. Stone, Philip McGuire, James H. MacCabe, Alice Egerton

**Affiliations:** 1 Department of Psychosis Studies, Institute of Psychiatry, Psychology & Neuroscience, King’s College London, De Crespigny Park, London, SE5 8AF UK; 2 Department of Psychiatry, Royal College of Surgeons in Ireland, Dublin, Ireland; 3 Department of Experimental Psychology, University of Oxford, Oxford, UK; 4 Department of Neuroimaging, Centre for Neuroimaging Sciences, Institute of Psychiatry, Psychology & Neuroimaging, King’s College London, De Crespigny Park, London, UK; 5 Experimental Medicine, Hammersmith Hospital, Imperial College London, London, UK

**Keywords:** Schizophrenia, n-Acetylcysteine, Magnetic resonance imaging, Resting state, Magnetic resonance spectroscopy, Glutamate, Functional connectivity

## Abstract

**Rationale:**

There is interest in employing N-acetylcysteine (NAC) in the treatment of schizophrenia, but investigations of the functional signatures of its pharmacological action are scarce.

**Objectives:**

The aim of this study was to identify the changes in resting-state functional connectivity (rs-FC) that occur following administration of a single dose of NAC in patients with schizophrenia. A secondary aim was to examine whether differences in rs-FC between conditions were mediated by glutamate metabolites in the anterior cingulate cortex (ACC).

**Methods:**

In a double-blind, placebo-controlled crossover design, 20 patients with schizophrenia had two MRI scans administered 7 days apart, following oral administration of either 2400 mg NAC or placebo. Resting state functional fMRI (rsfMRI) assessed the effect of NAC on rs-FC within the default mode network (DMN) and the salience network (SN). Proton magnetic resonance spectroscopy was used to measure Glx/Cr (glutamate plus glutamine, in ratio to creatine) levels in the ACC during the same scanning sessions.

**Results:**

Compared to the placebo condition, the NAC condition was associated with reduced within the DMN and SN, specifically between the medial pre-frontal cortex to mid frontal gyrus, and ACC to frontal pole (all *p* < 0.04). There were no significant correlations between ACC Glx/Cr and rs-FC in either condition (*p* > 0.6).

**Conclusions:**

These findings provide preliminary evidence that NAC can reduce medial frontal rs-FC in schizophrenia. Future studies assessing the effects of NAC on rs-FC in early psychosis and on repeated administration in relation to efficacy would be of interest.

**Electronic supplementary material:**

The online version of this article (10.1007/s00213-019-05382-1) contains supplementary material, which is available to authorized users.

## Introduction

There is an urgent need to develop new treatments for the large proportion of patients with schizophrenia who do not show an adequate response to standard antipsychotic treatment. Antipsychotics are predominantly dopamine D2 receptor antagonists (Nordström et al. 1993; Brisch 2014). This raises the possibility that patients who fail to respond to conventional antipsychotic treatment may benefit from mono- or adjunctive therapy with interventions that target non-dopaminergic mechanisms. In addition to dopamine dysfunction, schizophrenia is also associated with glutamatergic abnormalities (Moghaddam and Javitt 2012) (Merritt et al. 2016), functional dysconnectivity within brain networks (Stephan et al. 2006), inflammation (Khandaker et al. 2015) and oxidative stress (Flatow et al. 2013). Therefore, interventions that modulate these processes may also enhance treatment options for those who do not respond to conventional antipsychotics.

N-acetylcysteine (NAC) is an L-cysteine precursor (Arakawa and Ito 2007) and may have therapeutic potential due to its ability to regulate glutathione (GSH) and excessive brain glutamate through the cysteine-glutamate antiporter (Bridges et al. 2012), while increasing endogenous antioxidants and reducing inflammation (Berk et al. 2013).

NAC is under investigation as an adjunctive therapy for several psychiatric and neurological disorders (Deepmala et al. 2015). In schizophrenia, an initial double-blind clinical trial of NAC (1000 mg BD) over 24 weeks, administered adjunctive to antipsychotic medication, found a significant reduction in Positive and Negative Syndrome Scale (PANSS) negative symptom score (Berk et al. 2008). A subgroup of patients in this study who were receiving clozapine also showed significant improvements in negative, general and total scores at 8 weeks compared to the placebo group, although there was no significant group difference at the end of the trial (Dean et al. 2015). A clinical trial of NAC adjunct to risperidone in schizophrenia found reductions in PANSS total and negative symptom scores (Farokhnia et al. 2013). Moreover, recent clinical trials of NAC conducted in participants with early psychosis may also indicate efficacy. Administration of NAC (2700 mg/day over 6 months) resulted in improvements in neurocognition, although there was no significant effect on positive or negative symptoms or functional outcome (Conus et al. 2018), while a further study of NAC (3600 mg/day over 12 months) found significant improvement in PANSS total and negative scores and disorganised thought, but not positive symptoms or cognition (Breier et al. 2018).

While there is growing interest in the therapeutic potential of NAC in schizophrenia, there is limited information on its interactions with brain activity in man. We recently reported that a single dose of NAC compared to placebo was associated with lower levels of glutamate plus glutamine (Glx) in the anterior cingulate cortex (ACC) (McQueen et al. 2018), similar to findings in cocaine-addicted individuals (Schmaal et al. 2012). The clinical trial of Conus et al. (2018) examining the efficacy of NAC in early psychosis included investigation of central and peripheral biomarkers. While NAC did not affect glutamate in the medial frontal cortex over 6 months, GSH increased in this brain region, as did redox markers in peripheral blood. Repeated administration of NAC has also been shown to modulate EEG synchronisation (Carmeli et al. 2012) and improve mismatch negativity (Lavoie et al. 2008) in patients with schizophrenia. However, it is unknown whether modulation of brain activity can be observed following a single-dose administration, which may potentially provide an indication of target engagement for dose-finding, or as a biomarker to predict subsequent response.

Functional magnetic resonance imaging (fMRI) using Blood Oxygen Level Dependant (BOLD) signals can identify networks of correlated brain activity at rest (resting state functional connectivity, rs-FC). Schizophrenia is associated with rs-FC abnormalities across multiple networks and can thus be conceptualised as a disorder of ‘dysconnectivity’ (Stephan et al. 2006) although there is variance in pattern and directionality of the apparent dysconnections (Moran et al. 2013; Littow et al. 2015; Dong et al. 2017). This may relate to the stage of illness, with larger studies investigating the DMN within medication-naive first-episode psychosis predominantly reporting increases within and between network connectivity (Anticevic et al. 2013; Guo et al. 2014; Li et al. 2015). In contrast, decreased connectivity is principally found in larger studies in chronic schizophrenia (Meda et al. 2012, 2014), regardless of the methodology used (Pettersson-Yeo et al. 2011).

Rs-FC may also predict outcome in early-schizophrenia after treatment onset, as symptomatic improvement has been associated with normalisation of prefrontal cortex hyperconnectivity (Anticevic et al. 2015), and increased rs-FC in corticostriatal regions (Sarpal et al. 2015). Changes in brain network connectivity may reflect an emergent effect of glutamate signalling (Anticevic et al. 2015; Krystal et al. 2017), so may have relevance as biomarker for compounds that alter brain glutamate.

The main aim of the current study was to determine the effects of NAC on brain network resting state functional connectivity, in the same cohort of participants with schizophrenia in which we previously observed lower levels of ACC Glx following administration of NAC versus placebo. A secondary aim was to explore whether differences in rsfMRI between NAC and placebo administration were related to differences in ACC Glx levels.

## Methods

### Participants and clinical measures

This study had ethical approval from the NRES London-Harrow NHS ethics committee and was registered on clinicaltrials.gov (NCT02483130). As previously described (McQueen et al. 2018), the sample included 20 participants meeting DSM-IV criteria for schizophrenia, who were recruited from outpatient services within the South London and the Maudsley NHS Foundation Trust. Inclusion required written informed consent and good physical health, as determined by a physical health screen. Exclusion criteria included contraindications to MRI or NAC administration, including pregnancy, history of asthma, seizure and drug or alcohol dependency. Clinical symptom severity was assessed only on study entry, using the positive and negative syndrome scale for schizophrenia (PANSS) (Kay et al. 1982).

### Administration of N-acetyl cysteine and placebo

The order of NAC and placebo administration was block randomised such that an equal number of participants received either NAC or placebo in the first visit. Administration of 2400 mg NAC or placebo occurred 1 h before MRI scanning, so that scanning coincided with NAC peak plasma levels (Holdiness 1991). For each participant, the two MRI sessions occurred at the same time of day, 7 days apart. The local pharmacy distributed medication packs with visually identical capsules for each session. Both the research team and the participants were blind to the order and nature of each administration.

### Magnetic resonance imaging

MRI data were acquired on a 3-T MR750 scanner (General Electric, Chicago, USA). The scanning session commenced with a localizer, standard axial T2-weighted fast spin echo scan (TR/TE = 4380 ms/55.72 ms) and a 3D T1-weighted structural scan (TR/TE/TI = 7.312 ms/3.01 ms/400 ms; flip angle = 11^°^) with an isotropic spatial resolution of 1 mm.

The single-echo resting state fMRI sequence lasted 6 min and, employed an echo planar image acquisition (TR/TE = 2000 ms/30 ms, flip angle = 75^°^, field of view = 211 × 211 mm^2^, matrix = 64 × 64, slice thickness/gap = 3.0/0.3 mm, 40 oblique axial slices oriented parallel to the AC/PC line, 180 time points). For the duration of the acquisition, participants were instructed to keep their eyes open and look at a white fixation cross, presented on a black screen.

^1^H-MRS spectra were acquired in a 8-cm^3^ (2 × 2 × 2 cm^3^) voxel prescribed in the bilateral ACC, using a conventional PRESS (Point RESolved Spectroscopy) acquisition with 96 averages, TR = 3000 ms and with a TE = 30 ms in the ACC (McQueen et al. 2018). An additional 16 averages were acquired without water suppression for subsequent eddy current correction. The acquisition used the standard GE PROBE (PROton Brain Examination) sequence with CHESS (CHEmically Selective Suppression) water suppression.

### Image processing

Resting state fMRI data were analysed using the CONN Functional Connectivity Toolbox (http://web.mit.edu/swg/software.htm) (Whitfield-Gabrieli and Nieto-Castanon 2012), running in SPM12 (http://www.fil.ion.ucl.ac.uk/spm/), and Matlab 6.5 (Mathworks Inc. Sherbon, MA, USA). For image pre-processing within the CONN toolbox, T1-w images were segmented into grey matter, white matter and cerebrospinal fluid (CSF), and functional data was realigned and unwarped, slice-time corrected, co-registered to structural data, segmented, normalized to MNI space and spatially smoothed with an 8-mm at full-width half maximum (FWHM) three-dimensional Gaussian kernel using the standardised pipeline (Whitfield-Gabrieli and Nieto-Castanon 2012). The CompCor method (Behzadi et al. 2007) was used for isolation of the blood oxygen level dependant (BOLD) signal, scrubbing and band-pass temporal filtering (0.008–0.09 Hz) for noise reduction. Mean frame-wise displacement was calculated for all participants, and excessive movement excluded from second-level analysis. CONN’s quality assurance plots were assessed to identify any significant changes or displacement to global signal, subject motion or artefacts across all individual scans. ^1^H-MRS spectra were analysed with LCModel version 6.3-0I (Provencher 1993).

The full data relating to ^1^H-MRS study in this cohort is available in our previous publication (McQueen et al. 2018). As this study detected a significant difference in ACC Glx (glutamate + glutamine) scaled to creatine (Glx/Cr) between the placebo and NAC conditions (but not in glutamate/Cr or in glutamate or Glx corrected for voxel tissue composition), in the current study, we applied the same ACC Glx/Cr measure to investigate the relationship with changes in rs-FC.

### Statistical analysis

To identify differences in resting state functional connectivity between the placebo and NAC conditions, analysis examined rs-FC between 11 seed ROIs from within the Default Mode Network (DMN) or Salience Network (SN) and target ROI’s including 106 cortical or subcortical regions to create a 11 × 106 connectivity matrix (Supplement Figure 1). Seed ROI (with MNI coordinates) within the DMN included the medial prefrontal cortex (MPFC, 1, 55, − 3), lateral parietal (L) cortex (− 39, − 77, 33), lateral parietal cortex (R) (47, − 67, 29), posterior cingulate cortex (1, − 61, 38)), and seed ROI within the SN included the anterior cingulate cortex (ACC, 0, 22, 35), anterior insula cortex (L) (− 44, 13, 1), anterior insula cortex (R) (47, 14, 0), rostral prefrontal cortex (L) (− 32, 45, 27), rostral prefrontal cortex (R) (32, 46, 27), supramarginal gyrus (L) (− 60, − 39, 31) and supramarginal gyrus (R) (62, − 35, 32). At the first-level, ROI-to-ROI Fisher-transformed correlation matrices were computed for each subject. At the second level, sessions (NAC, placebo) were contrasted to determine the connectivity differences after NAC or placebo administration. Significant differences were accepted at *p* < 0.05 with FDR correction to control for multiple comparisons.

**Fig. 1:**
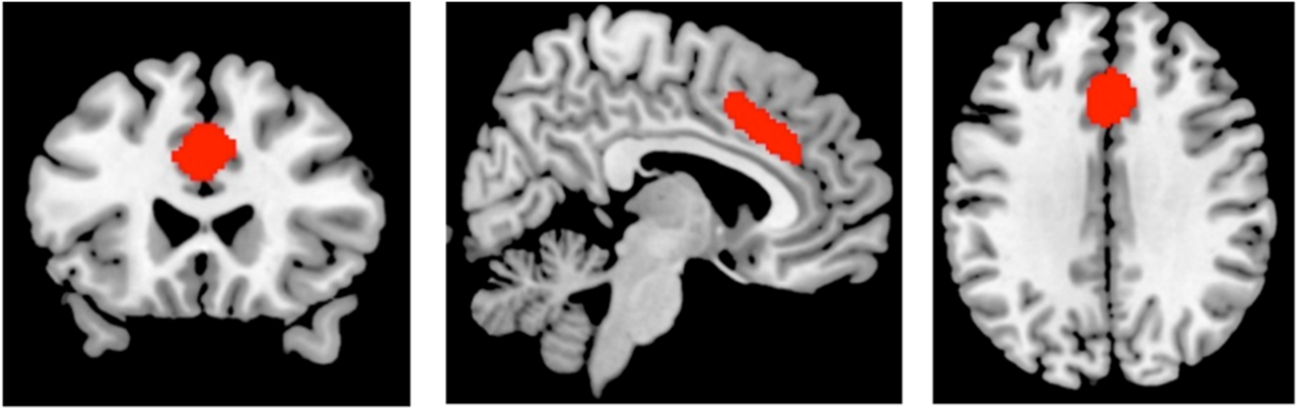
Resting state network seed region of interest in the anterior cingulate gyrus used for the covariate analysis with glutamate and glutamine scaled to creatine. The seed is preselected from the ROI > Networks menu within CONN during the first level analysis.

To determine the relationships between glutamate (measured as Glx/Cr) in the ACC and rs-FC under the placebo and NAC conditions, we performed a seed-to-voxel analysis, utilising an ACC network seed ROI (MNI: 0, 22, 35; Fig. 1) and adding individual ACC Glx/Cr values as second level covariates. At the second level, we examined whether ACC seed connectivity covaried with ACC Glx/Cr in both the NAC and placebo sessions.

## Results

### Sample characteristics

Demographic and clinical measures are presented in Table 1. Datasets were available in 17 of the 20 participants as one patient experienced an adverse reaction after NAC administration, one participant did not complete a full scan due to limited scanning time and one dataset was not retrieved due to a fault with the scanner. No data were excluded due to excessive movement (resting state mean motion ± standard deviation = 0.23 ± 0.11). As detailed previously (McQueen et al. 2018), participants were receiving antipsychotic medication from 4 months to 19 years (mean (SD) months = 83.69 (71.30)) and had an illness duration of 4 months to 30 years (mean (SD) months = 152.00 (93.56). No participant was taking any illicit drugs during the study.

**Table 1:** Subject demographics and clinical measures

Demographic and clinical statistics	Participants (*N* = 17)
Age, years	41.28 (11.23)
Gender, female/male	3/14
Handedness, right/left	17/0
PANSS–positive	12.69 (4.37)
PANSS–negative	14.05 (3.96)
PANSS–general	23.37 (6.40)
PANSS–total	50.12 (12.91)
Antipsychotic medication–Ol/Ri/Ar/Pa/Ha/Zu/Fl	7/4/2/2/1/1/1

Data are reported as mean (standard deviation)

*PANSS* Positive and Negative Syndrome Scale scores, *Ar* aripiprazole, *Fl* flupentixol, *Ha* haloperidol, *Ol* olanzapine, *Pa* paliperidone, *Ri* risperidone, *Zu* zuclopenthixol

### Effect of NAC on resting state functional connectivity

**Fig. 2:**
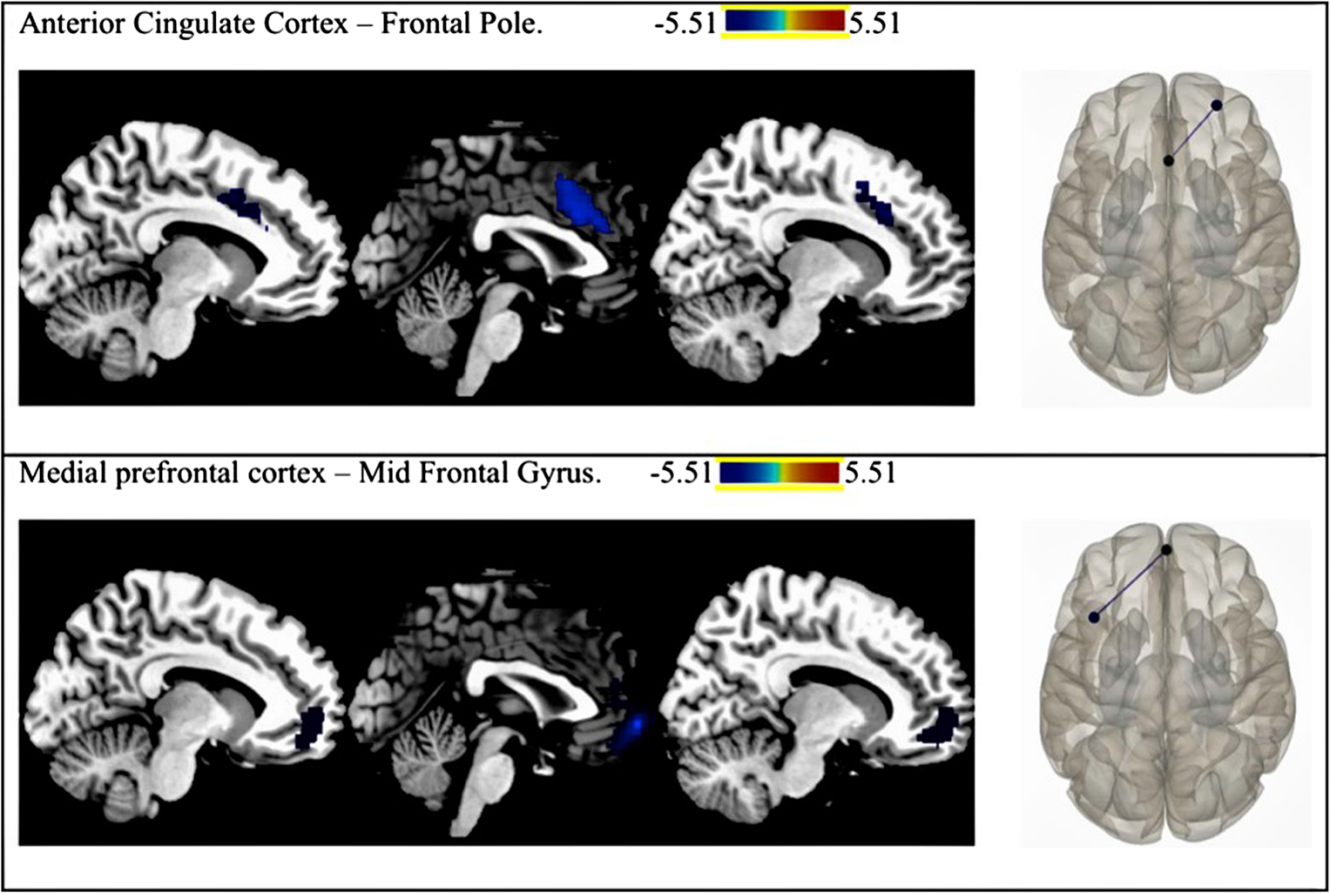
Differences in resting state functional connectivity following a single administration of 2400 mg NAC versus placebo in patients with schizophrenia, in the salience network (top panel) and default mode network (bottom panel). The colour bar represents the *t* value. In the NAC compared to placebo condition, rs-FC was significantly lower between the anterior cingulate cortex (0, 22, 35) and frontal pole (26, 52, 8) and between the medial prefrontal cortex (1, 55, − 3) and mid frontal gyrus (− 38, 18, 42) (*p <* 0.05, FDR corrected). Results are overlaid on sagittal T1-weighted MRI images (left) (Holmes et al. 1998; Rorden et al. 2007), and complementary axial images generated in CONN (right) display the connectivity between the seed and target ROIs.

**Fig. 3:**
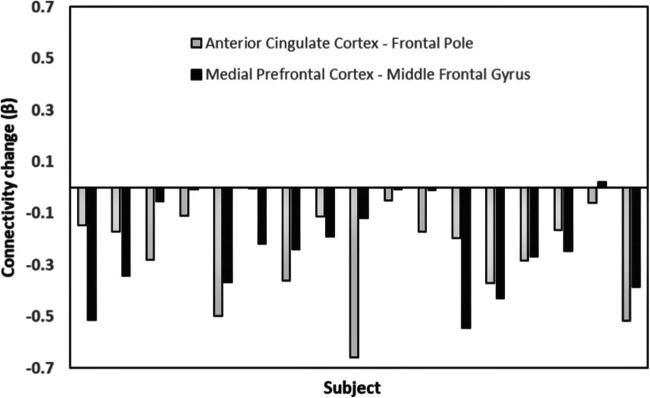
Individual subject differences in resting state functional connectivity following a single administration of 2400 mg NAC versus placebo in the default mode network and salience network. Lower levels of connectivity in the NAC compared to placebo condition were apparent in all subjects between the anterior cingulate cortex to frontal pole, and 16/17 subjects in the medial prefrontal cortex to middle frontal gyrus.

Compared to placebo, the NAC condition was associated with lower levels of connectivity within the DMN and SN (Table 2). This was apparent between the medial prefrontal cortex and mid-frontal gyrus in the DMN, and ACC and frontal pole in the SN (all *p* < 0.04, Fig. 2). The differences in rs-FC in the NAC compared to placebo conditions in individual subjects are presented in Fig. 3.

**Table 2:** Reductions in resting state functional connectivity following administration of 2400 mg N-acetylcysteine compared to placebo in the Default Mode (DMN) and Salience networks (SN) in patients with schizophrenia. Node and Montreal Neurological Institute (MNI) coordinates of regions of interest with significant *p* values (FDR corrected, controlling for multiple comparisons).

Node and MNI coordinate	Network	*p* FDR	*t* statistic
Medial prefrontal cortex (1, 55, − 3)–mid frontal gyrus (− 38, 18, 42) Anterior cingulate cortex (0, 22, 35)–frontal pole (26, 52, 8)	DMN	0.04	*t* (16) = − 4.75
	SN	0.01	*t* (16) = − 5.51

### Relationship between resting state functional connectivity and Glx

As previously reported, the mean ± standard deviation of ACC Glx/Cr in the placebo condition was 1.86 ± 0.43, and in the NAC condition was 1.63 ± 0.30 (McQueen et al. 2018). Spectral quality did not differ between conditions (McQueen et al. 2018). There were no significant correlations in our seed-to-voxel analysis between ACC Glx/Cr and rs-FC in either the placebo or the NAC condition (all *p* > 0.6).

## Discussion

This study evaluated brain network rs-FC following a single dose of NAC versus placebo in patients with schizophrenia. In the NAC versus placebo condition, rs-FC differences were apparent in frontal areas within the DMN (between the medial prefrontal cortex and frontal gyrus) and SN (anterior cingulate gyrus and frontal pole). These differences did not appear to be related to ACC glutamatergic metabolite levels.

The observed differences in connectivity within the DMN and SN under the NAC compared to placebo conditions provide preliminary evidence that NAC administration can modulate connectivity within brain functional networks, including those implicated in schizophrenia (Pettersson-Yeo et al. 2011; Dong et al. 2017). Activity within the DMN reflects self-monitoring and thoughts independent of stimuli (Buckner et al. 2008), and DMN dysconnectivity in schizophrenia may be indicative of a failure to identify thoughts as internally generated (Frith 1995). The SN is involved in evaluating the homeostatic or emotional importance of external stimuli for further processing or action (Seeley et al. 2007; Sridharan et al. 2008; Medford and Critchley 2010). Both dysconnectivity within the DMN and SN have been associated with the severity of positive symptoms including delusions or hallucinations, as well as negative symptoms, emotional and cognitive dysfunction (Palaniyappan et al. 2012, 2013; Peters et al. 2016; Sheffield and Barch 2016; Lee et al. 2018).

In schizophrenia, there is an overall profile of decreased connectivity, particularly affecting anterior cingulate or medial prefrontal cortex connectivity (Pettersson-Yeo et al. 2011; Dong et al. 2017). However, there is some indication that this hypoconnectivity is more marked in older groups at more chronic stages of illness (Dong et al. 2017), and that early psychosis may be associated with frontal hyperconnectivity (Guo et al. 2014; Anticevic et al. 2015; Li et al. 2015). Within this context, our findings indicating that acute NAC reduces frontal rs-FC may indicate that NAC might be more effective in attenuating hyperconnectivity and potentially improving functioning in early stages of psychosis but could worsen hypoconnectivity at later stages of illness. This speculative interpretation could be tested by comparing the effects of NAC on rs-FC in patients at different illness stages. While the current manuscript was under review, a pilot study reported increases in cingulate rs-FC after 6 months of administration NAC compared to placebo in patients with early psychosis (Mullier et al. 2019). In participants undergoing nicotine withdrawal, 3 days of NAC treatment also increased connectivity in DMN nodes (specifically the mPFC) (Froeliger et al. 2015). The contrast between these findings and the lower levels of frontal connectivity that were associated with the NAC condition in our study may suggest differences in rs-FC emerging on repeated compared to single-dose NAC administration.

In our seed-to-voxel analysis, we did not identify any correlations between ACC Glx/Cr and rs-FC of the ACC seed after either NAC or placebo administration. This may suggest that the reduction in Glx/Cr in the NAC condition reported previously (McQueen et al. 2018) is not directly correlated with changes in ACC rs-FC, or that we were limited to detect this association within our study design. Other studies combining ^1^H-MRS and rs-FC find relationships between rs-FC and regional glutamate or GABA levels (Duncan et al. 2014). A previous study in healthy volunteers detected a positive correlation between frontal rs-FC and medial prefrontal cortex glutamate levels (Duncan et al. 2013); however, a study comparing patients with schizophrenia to healthy volunteers found no association in glutamate and GABA concentrations and differences in functional connectivity (Shukla et al. 2018). In contrast, Falkenberg and colleagues reported a positive association between glutamate levels and parietal FC in patients with schizophrenia, while these measures were negatively correlated in healthy volunteers (Falkenberg et al. 2014). The absence of a significant association between ACC Glx/Cr and rs-FC in our study may reflect the limitations of the ^1^H-MRS approach in measuring glutamate that is involved in neurotransmission specifically, or other experimental limitations such as sample size. Changes in Glx/Cr will reflect both the numerator and denominator, as more fully discussed in our previous manuscript (McQueen et al. 2018). In addition, inclusion of a healthy volunteer cohort would have strengthened our study by allowing investigation of whether the normal relationship between glutamate metabolites and rs-FC was altered in our patient sample.

As participants in this study were blind to the order of placebo and NAC administration, potential effects of expectation of active drug effects on rs-FC were minimised. The study was performed in participants with schizophrenia as we were interested in the interaction of NAC with pathological network activity. As these effects were largely unknown, we employed an exploratory whole-brain analysis. The CONN toolbox incorporates several techniques which are known to reduce confounds while increasing the clarity of findings, including the anatomical CompCor approach in order to improve the signal-to-noise ratio in fMRI scans (Whitfield-Gabrieli and Nieto-Castanon 2012). This method extracts principal components from white matter and cerebrospinal fluid time series during segmentation and includes these components in the denoising step as confounds. The CompCor method shows a high degree of inter-scan reliability, while also increasing both the selectivity, and the sensitivity of connectivity findings (Whitfield-Gabrieli and Nieto-Castanon 2012). Our study recruited a general sample of medicated participants with established schizophrenia. Investigation of the effects of NAC in comparison to those in healthy volunteers or anti-psychotic-free patients with early psychosis would be of future interest, as would be investigation of the effects of longer-term NAC administration on rs-FC in relationship to clinical outcome. Sample size may have limited the ability to detect additional differences in rs-FC between conditions and relationships with ACC Glx, and while data acquisition was timed to coincide with peak NAC plasma levels (Holdiness 1991), we did not investigate the time-course of effects.

In summary, this study showed a single dose of NAC was associated with decreases in rs-FC in prefrontal cortical regions of the DMN and SN network in patients with established schizophrenia. While the effects of repeated administration of NAC on rs-FC in schizophrenia remain to be evaluated, our results may imply that NAC could reduce frontal hyperconnectivity that has been more strongly associated with the earlier illness stages (Sarpal et al. 2015; Anticevic et al. 2015). Recent clinical trials have found some indications of efficacy of NAC in early psychosis (Conus et al. 2018; Breier et al. 2018), which may be mediated by baseline redox status (Conus et al. 2018) or cortical structural integrity (Breier et al. 2018). The relationships between these biomarkers in addition to rs-FC and glutamate measures (Egerton et al. 2018; McQueen et al. 2018) and improvements in symptoms could be tested in future clinical trials of NAC or similar compounds.

## Electronic supplementary material

ESM 1 (DOCX 490 kb)
