# Supplementary material for: Effect of single dose N-acetylcysteine administration on resting state functional connectivity in schizophrenia
Source: Psychopharmacology (Berl). 2019 Nov 30;237(2):443–51. doi: 10.1007/s00213-019-05382-1 (PMC7018675; doi:10.1007/s00213-019-05382-1)
Supplement: Supplementary file 1 — (DOCX 490 kb) [file 213_2019_5382_MOESM1_ESM.docx]

**McQueen *et al.,* Effect of single dose N-acetylcysteine administration on resting state functional connectivity in schizophrenia.**


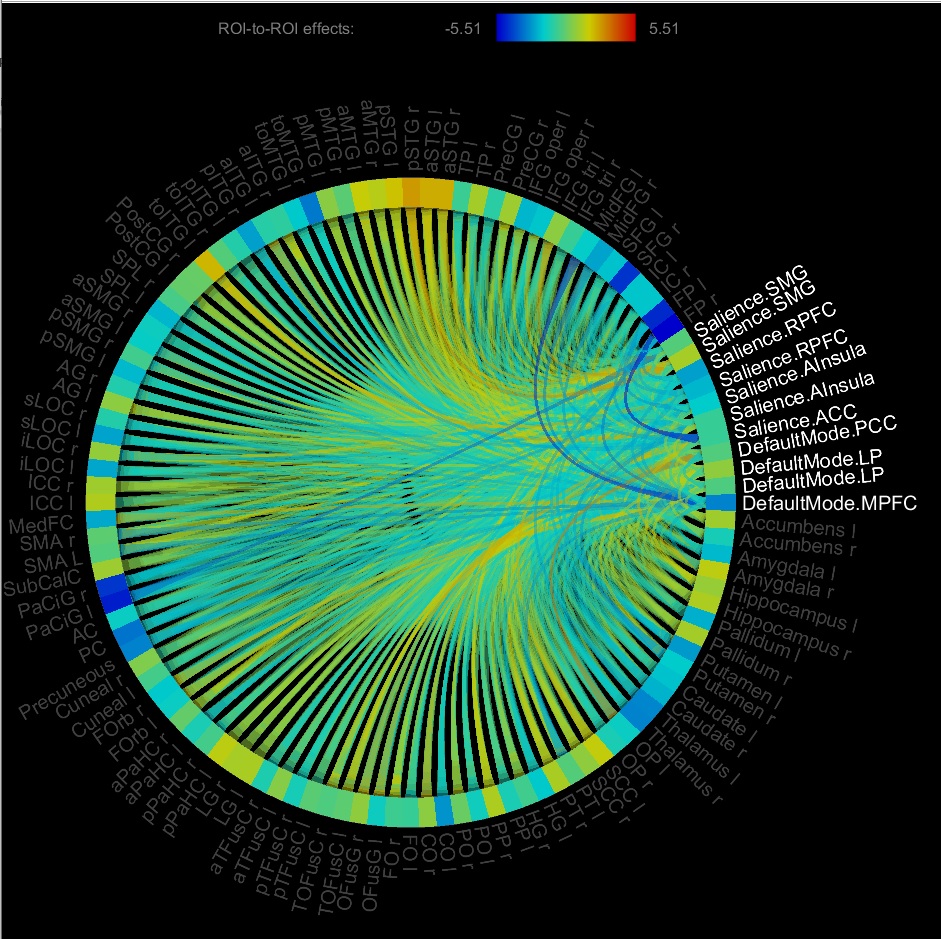
Supplement Figure 1

**Supplement Figure 1.** Connectome ring produced in CONN displaying the ROI to ROI rs-FC matrix between eleven seed ROI’s within the salience and default mode networks and 106 cortical or subcortical ROIs. The colour bar represents the t value. In the NAC compared to placebo condition, rs-FC was significantly lower between the medial pre-frontal cortex and mid frontal gyrus, and between the ACC and frontal pole (*p <* 0.05, FDR corrected).

**Abbreviations**: FP r (Frontal Pole Right); FP l (Frontal Pole Left); IC r (Insular Cortex Right); IC l (Insular Cortex Left); SFG r (Superior Frontal Gyrus Right); SFG l (Superior Frontal Gyrus Left); MidFG r (Middle Frontal Gyrus Right); MidFG l (Middle Frontal Gyrus Left); IFG tri r (Inferior Frontal Gyrus, pars triangularis Right); IFG tri l (Inferior Frontal Gyrus, pars triangularis Left); IFG oper r (Inferior Frontal Gyrus, pars opercularis Right); IFG oper l (Inferior Frontal Gyrus, pars opercularis Left); PreCG r (Precentral Gyrus Right); PreCG l (Precentral Gyrus Left); TP r (Temporal Pole Right); TP l (Temporal Pole Left); aSTG r (Superior Temporal Gyrus, anterior division Right); aSTG l (Superior Temporal Gyrus, anterior division Left); pSTG r (Superior Temporal Gyrus, posterior division Right); pSTG l (Superior Temporal Gyrus, posterior division Left); aMTG r (Middle Temporal Gyrus, anterior division Right); aMTG l (Middle Temporal Gyrus, anterior division Left); pMTG r (Middle Temporal Gyrus, posterior division Right); pMTG l (Middle Temporal Gyrus, posterior division Left); toMTG r (Middle Temporal Gyrus, temporooccipital part Right); toMTG l (Middle Temporal Gyrus, temporooccipital part Left); aITG r (Inferior Temporal Gyrus, anterior division Right); aITG l (Inferior Temporal Gyrus, anterior division Left); pITG r (Inferior Temporal Gyrus, posterior division Right); pITG l (Inferior Temporal Gyrus, posterior division Left); toITG r (Inferior Temporal Gyrus, temporooccipital part Right); toITG l (Inferior Temporal Gyrus, temporooccipital part Left); PostCG r (Postcentral Gyrus Right); PostCG l (Postcentral Gyrus Left); SPL r (Superior Parietal Lobule Right); SPL l (Superior Parietal Lobule Left); aSMG r (Supramarginal Gyrus, anterior division Right); aSMG l (Supramarginal Gyrus, anterior division Left); pSMG r (Supramarginal Gyrus, posterior division Right); pSMG l (Supramarginal Gyrus, posterior division Left); AG r (Angular Gyrus Right); AG l (Angular Gyrus Left); sLOC r (Lateral Occipital Cortex, superior division Right); sLOC l (Lateral Occipital Cortex, superior division Left); iLOC r (Lateral Occipital Cortex, inferior division Right); iLOC l (Lateral Occipital Cortex, inferior division Left); ICC r (Intracalcarine Cortex Right); ICC l (Intracalcarine Cortex Left); MedFC (Frontal Medial Cortex); SMA r (Juxtapositional Lobule Cortex -formerly Supplementary Motor Cortex- Right); SMA L(Juxtapositional Lobule Cortex -formerly Supplementary Motor Cortex- Left); SubCalC (Subcallosal Cortex); PaCiG r (Paracingulate Gyrus Right); PaCiG l (Paracingulate Gyrus Left); AC (Cingulate Gyrus, anterior division); PC (Cingulate Gyrus, posterior division); Precuneous (Precuneous Cortex); Cuneal r (Cuneal Cortex Right); Cuneal l (Cuneal Cortex Left); FOrb r (Frontal Orbital Cortex Right); FOrb l (Frontal Orbital Cortex Left); aPaHC r (Parahippocampal Gyrus, anterior division Right); aPaHC l (Parahippocampal Gyrus, anterior division Left); pPaHC r (Parahippocampal Gyrus, posterior division Right); pPaHC l (Parahippocampal Gyrus, posterior division Left); LG r (Lingual Gyrus Right); LG l (Lingual Gyrus Left); aTFusC r (Temporal Fusiform Cortex, anterior division Right); aTFusC l (Temporal Fusiform Cortex, anterior division Left); pTFusC r (Temporal Fusiform Cortex, posterior division Right); pTFusC l (Temporal Fusiform Cortex, posterior division Left); TOFusC r (Temporal Occipital Fusiform Cortex Right); TOFusC l (Temporal Occipital Fusiform Cortex Left); OFusG r (Occipital Fusiform Gyrus Right); OFusG l (Occipital Fusiform Gyrus Left); FO r (Frontal Operculum Cortex Right); FO l (Frontal Operculum Cortex Left); CO r (Central Opercular Cortex Right); CO l (Central Opercular Cortex Left); PO r (Parietal Operculum Cortex Right); PO l (Parietal Operculum Cortex Left); PP r (Planum Polare Right); PP l (Planum Polare Left); HG r (Heschl's Gyrus Right); HG l (Heschl's Gyrus Left); PT r (Planum Temporale Right); PT l (Planum Temporale Left); SCC r (Supracalcarine Cortex Right); SCC l (Supracalcarine Cortex Left); OP r (Occipital Pole Right); OP l (Occipital Pole Left); Thalamus r; Thalamus l; Caudate r; Caudate l; Putamen r; Putamen l; Pallidum r; Pallidum l; Hippocampus r; Hippocampus l; Amygdala r; Amygdala l; Accumbens r; Accumbens l.
